# Genome-Wide Profiling of MicroRNAs in Adipose Mesenchymal Stem Cell Differentiation and Mouse Models of Obesity

**DOI:** 10.1371/journal.pone.0021305

**Published:** 2011-06-23

**Authors:** Lena Bengestrate, Sam Virtue, Mark Campbell, Antonio Vidal-Puig, Dirk Hadaschik, Peter Hahn, Wolfgang Bielke

**Affiliations:** 1 Department of Epigenetics, QIAGEN GmbH, Hilden, Germany; 2 Metabolic Research Laboratories, Institute of Metabolic Science, Addenbrooke's Hospital, University of Cambridge, Cambridge, United Kingdom; University of Barcelona, Spain

## Abstract

In recent years, there has been accumulating evidence that microRNAs are key regulator molecules of gene expression. The cellular processes that are regulated by microRNAs include e.g. cell proliferation, programmed cell death and cell differentiation. Adipocyte differentiation is a highly regulated cellular process for which several important regulating factors have been discovered, but still not all are known to fully understand the underlying mechanisms. In the present study, we analyzed the expression of 597 microRNAs during the differentiation of mouse mesenchymal stem cells into terminally differentiated adipocytes by real-time RT-PCR. In total, 66 miRNAs were differentially expressed in mesenchymal stem cell-derived adipocytes compared to the undifferentiated progenitor cells. To further study the regulation of these 66 miRNAs in white adipose tissue *in vivo* and their dependence on PPARγ activity, mouse models of genetically or diet induced obesity as well as a mouse line expressing a dominant negative PPARγ mutant were employed.

## Introduction

During the last decades, obesity and associated diseases, such as type 2 diabetes, hypertension, and cardiovascular disease, have reached an epidemiologic proportion [1], [2]. This fact has led to intensive studies, conducted with the help of suitable cell culture systems, supplemented by transgenic- or knock-out mouse models to investigate the genetic or environmental factors influencing obesity. The employment of such mouse models has revealed more than 200 genes involved in the regulation of fat metabolism and adiposity. These genes may affect the regulation of food uptake, differentiation of preadipocytes, or fat storage capability of adipocytes [3]. Although the role of many protein-encoding genes has been well established, much less is known about the regulatory influence of microRNAs (miRNAs) on proteins or gene-transcripts, involved in these processes. microRNAs are defined as a class of short interfering RNAs, which are capable to bind to partially complementary sequences localized within the 3′-untranslated regions (3′-UTRs) of mRNAs. miRNAs affect gene expression as part of a multi-modal protein complex, called RNA Induced Silencing Complex (RISC). Binding of miRNAs to target mRNAs results in translational repression or degradation of the targeted transcript. Cumulating evidence allocates miRNA functions to integral parts of regulatory cellular processes, including the differentiation of stem cell populations, the regulation of metabolic activities, and tumorigenesis [4], [5], [6].

Multipotent adult mesenchymal stem cells (MSC) are capable of differentiating into a variety of cell types, including adipocytes, osteoblasts, chondrocytes, and myocytes [7], [8], [9]. Regulation of stemcellness vs. differentiation is a key event in mesenchymal stem cell biology, and allows appropriate tissue homeostasis, while maintaining a sufficient pool of precursor cells for further use. Such pools of mesenchymal stem cells, residing in the bone marrow or the vascular stroma of adipose tissue, are also the source for MSCs, which ultimately develop into adult adipocytes [10], [11], [12]. *In vitro*, MSCs proliferate and develop into preadipocytes after reaching confluency. Preadipocytes are still competent for proliferation but, when allowed to become confluent and treated with substances such as insulin and glucocorticoids, cease cell division and differentiate into adipocytes, [13]. At the molecular level, the core process of adipogenesis is regulated by transcription factors, such as CCAAT/enhancer binding protein α (C/EBP-α) and peroxisome proliferator activated receptor γ (PPARγ), a heterodimerization partner of Retinoic X receptors (RXRs) [14], [15], [16]. The concerted action of these adipogenic transcription factors ultimately drives the expression of adipocyte specific factors such as enzymes responsible for the synthesis and storage of triglycerides in lipid droplets. [17], [18].

Here, we describe the results of a miRNome-wide analysis of primary adipocyte differentiation, conducted by a novel screening strategy. By using pools of RNAs from MSCs at different time points after adipocyte differentiation, we could reduce the complexity of such a real-time PCR based screening approach while keeping the detection sensitivity. Pools of 5 RNAs from undifferentiated MSCs or differentiated cells at four different time points were used for RT-PCR analysis in order to exclude those miRNAs from further tests which were not present at detectable levels in any of the samples. In a second step, detectable miRNA species were investigated in individual RNA samples for their expression patterns during *in vitro* differentiation of MSC. Regulated miRNA candidates, revealed during the MSC screen, were further studied *in vivo* using white fat tissue samples from mouse models of genetically or diet induced obesity. Whether the expression of these miRNAs was dependent on PPARγ activity *in vivo* was further analyzed in a mouse model harbouring a dominant-negative PPARγ mutation (P465L).

## Materials and Methods

### Cell culture

All cells were cultured in a 5% CO_2_ humified atmosphere at 37°C in T25-culture flasks. The culture media were changed every second or third day. Mouse mesenchymal stem cells (MSC) derived from bone marrow of 4 weeks old C57/Bl6 mice were a kind gift from the group of Viktor Wixler, University of Münster. The cells were previously characterized as mesenchymal stem cells by determination of surface marker genes using quantitative RT-PCR and their potential to differentiate into osteogenic, chondrogenic and adipogenic lineages [19]. The MSC were cultivated in a mixture of 60% DMEM and 40% MCDB-201 medium (Sigma-Aldrich) supplemented with 2% FCS, 100 units penicillin/ml, 100 µg/ml streptomycin, 10 ng/ml EGF and 10 ng/ml PDGF (R&D Systems), 1 mg/ml linoleic acid (Sigma-Aldrich), 10 ng/ml leukemia inhibitory factor (LIF, Chemicon International, Inc.), 5 µg/ml insulin-transferrin-selenium mixture (Sigma-Aldrich), 0.1 mM ascorbate-2-phosphate and 10^−2^ µM dexamethasone (DMX) (Sigma-Aldrich).

MSC were induced to differentiate at the third day after reaching confluency by addition of differentiation medium (60% DMEM, 40% MCDB-201, 2% FCS, 100 units penicillin/ml,100 µg/ml streptomycin ,1 mg/ml linoleic acid, 0.1 mM ascorbate-2-phosphate, 0.5 mM 3-isobutyl-1-methylxanthine (IBMX), 1 µM DMX and 10 µg/ml insulin-transferrin-selenium mixture). After three days the medium was changed to adipocyte maintenance medium containing the same supplements as the differentiation medium but with hundred fold lower DMX concentration and lacking IBMX. The cells were cultivated for additional 7 days. Total RNA including miRNA was isolated from normal proliferating cells (as reference), as well as from confluent cells at the day of induction (d_0_), and at two (d_2_), four (d_4_), seven (d_7_) and ten (d_10_) days after induction using miRNeasy Mini Kit according to the manufacturers protocol (QIAGEN). For verification of the results, cells from two passages (passages number 5 and 8) were analyzed independently. For visualisation of the differentiated adipocytes, the cells were stained with Oil Red O by washing twice with PBS and fixing with 4% paraformaldehyde in PBS at 4°C over night. The solution was removed and the fixed cells were washed with 60% isopropanol. After drying, cells were stained in fresh Oil Red O solution (60% Oil Red O stock, consisting of 0.35% Oil Red O in isopropanol, and 40% H_2_O) for at least one hour at room temperature. Subsequently, the cells were washed several times with water.

NIH/3T3-cells (American Type Culture Collection, ATCC) were cultivated in DMEM (Gibco) supplemented with 10% FCS (BioChrom), 100 units penicillin/ml,100 µg/ml streptomycin and non-essential amino acids (Invitrogen). After reaching confluency (k_0_) the cells were cultivated for further three days (k_1_–k_3_) and total RNA was isolated at every time point using miRNeasy Mini Kit according to the manufacturer's protocol (QIAGEN).

### Mouse studies

Unless stated otherwise, mice were housed in a temperature-controlled room (22°C) with a 12-hour light/dark cycle and had free access to food and water. All animal protocols were approved by the UK Home Office, licence number 80/2098 and the University of Cambridge. The first model consisted of leptin-deficient ob/ob mice on a C57BL/6 genetic background. ob/ob mice were fed a standard laboratory chow diet (10% calories from fat) and were compared to littermate wt control animals. The second group of mice consisted of animals exposed to diet-induced obesity. Mice on a mixed C57BL/6J/SV129 genetic background were fed a high-fat diet (HFD) for 10 weeks from weaning (45% calories from fat) and compared to littermate controls fed a chow diet from weaning. The final experimental group was a mouse model harbouring a dominant-negative PPARγ mutation (P465L) fed either a chow diet or HFD. Leptin-deficient ob/ob mice and litter mate controls were purchased from Harlan (Harlan, UK). Animals for the HFD studies and P465L DN mice were bred in-house at Cambridge University. For every model the epididymal fat pads of five 10–13 weeks old male mice were harvested, frozen in liquid nitrogen and stored at −80°C. Total RNA including miRNAs of all tissue samples were extracted using miRNeasy Mini Kit (QIAGEN) according to the manufacturer's protocol and the TissueRuptor (QIAGEN) for homogenisation.

### Quantitative real-time PCR

For mRNA analyses 100 ng total RNA were reverse-transcribed by Omniscript RT Kit (QIAGEN). For real-time PCR amplification QuantiTect SYBR Green PCR Kit and QuantiTect Primer Assays were used according to manufacturer's protocol (QIAGEN). GAPDH was used as endogenous control.

For miRNA analyzes 100 ng total RNA were reverse-transcribed and amplified in real-time PCR using miScript-System including miScript RT-Kit, miScript SYBR-Green PCR-Kit and miScript Primer Assay miRBase v12 (QIAGEN) according to the manufacturer's protocol. For internal control the expression of the small nuclear RNAs RNU1A, RNU6B and RNU5A as well as the expression of the small nucleolar RNAs SNORA73A, SNORD25, and SCARNA17 was determined. All PCR reactions were performed in triplicate in 384-well plates and measured by ABI 7900HT (Applied Biosystems). Mean values and standard deviations were calculated and fold changes were determined using the ΔΔC_T_ method. miRNAs displaying a fold change >2 or <0.5 during adipogenesis of mesenchymal stem cells or a fold change >1.5 or <0.66 in the *in vivo* obesity models in combination with p-values<0.05 were regarded as regulated. During the melting curve analysis after completion of the PCR amplification cycles, the temperature is increased slowly from 65°C to 95°C. At low temperatures all PCR products are double stranded and SYBR Green intercalates into these molecules leading to a high fluorescence signal. Whereas at high temperatures, PCR products are denatured, resulting in rapidly decreasing fluorescence signals. The fluorescence is measured continuously as the temperature rises and plotted against temperature. A curve is produced, as the fluorescence decreases slightly in the lower temperature range, but decreases much more rapidly at higher temperatures when the melting temperatures (Tm) of specific and nonspecific PCR products are reached. The detection system calculates the first derivatives of these curves, resulting in plots peaking at the respective specific melting temperature of this PCR product (Tm). These Tms may be used to check the specificity of the resulting PCR products.

### Agarose gel electrophoresis

After real-time PCR, the samples were analyzed by 2% agarose gel electrophoresis containing 0.3 µg/ml ethidium bromide at 100 V for 1 h in order to identify the amplicon sizes of the PCR products. As molecular weight marker the GenPilot 50 bp Ladder (QIAGEN) was used.

### Statistical analysis

For the statistical analysis of the expression data the student's t test was performed and p-values were calculated using the unpaired two-tails method in Excel (Microsoft). Fold changes with p-values<0.05 were considered to be statistically significant, and fold changes with p-values<0.01 to be highly significant. For the P465L DN mouse study results were analysed by two-way ANOVA using SPSS 17.0 (IBM, New York). Data were considered to be significant if there was either an effect of diet or genotype or if a diet*genotype interaction with a p-value<0.05 was observed. For the analysis of the 66 miRNAs we corrected for False Discovery Rate was corrected for using q statistics calculated using R (R Foundation). P values were accepted as valid if the Q statistic was less than P = 0.05.

## Results

### Adipocyte-differentiation of murine mesenchymal stem cells

**Figure 1:**
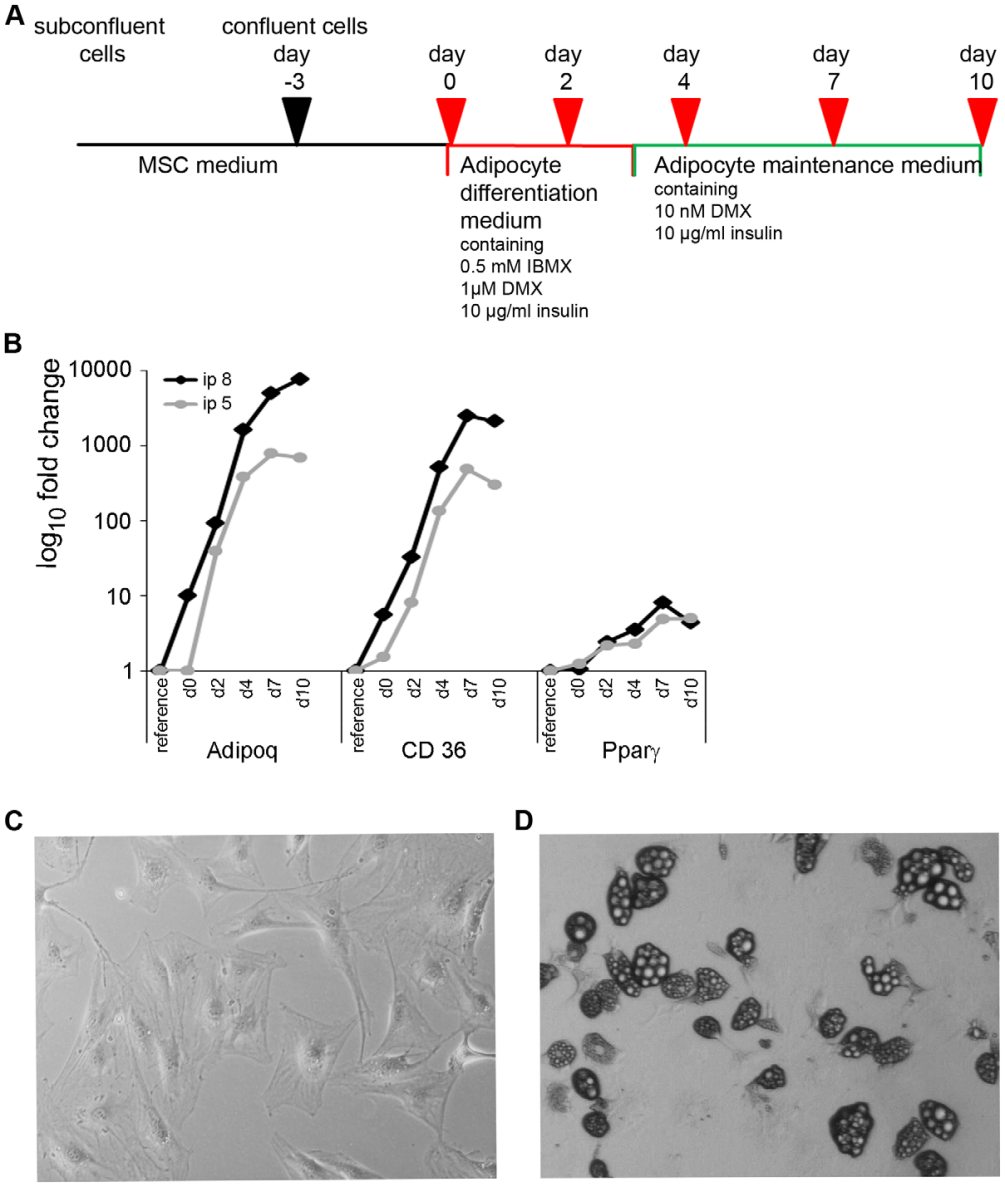
Differentiation of mesenchymal stem cells to adipocytes. **A**: Protocol for the differentiation of mesenchymal stem cells to adipocytes. **B**: Real-time PCR-based expression analysis of the adipocyte marker genes adiponectin (Adipoq), CD36 and PPARγ during adipogenesis of murine mesenchymal stem cells. For verification two cell passages (ip 5 and ip 8) were independently differentiated and analyzed. Data were normalized by GAPDH expression and plotted as fold change (log_10_ scale) relative to the expression levels of the undifferentiated subconfluent reference cells. **C, D**: Phenotypical change from fibroblast-like murine mesenchymal stem cells (C, 100× magnification) to round-shaped adipocytes (D, 40× magnification).

For this study, bone marrow-derived murine mesenchymal stem cells from 4 week-old C57BL/6 mice, that have been prepared by depletion of CD45-expressing cells and that have been shown to express CD34, c-kit, sca1 and CD13 [19], were used as the adipogenesis model. Adipogenic differentiation was induced as illustrated in Fig. 1A. Briefly, cells were cultivated until they reached confluency. Then cells were grown for an additional three days to induce growth arrest and subsequently the normal culture medium was changed to adipocyte differentiation medium (day 0). After cultivating the cells for further three days medium was changed to maintenance medium (at day 3) which subsequently was renewed every two days. Samples were taken from subconfluent cell culture (as a reference), at day 0, and 2, 4, 7 and 10 days after induction of adipocyte differentiation. The differentiation progress of two independent cell batches (referred to as passage 5 and passage 8) was monitored by measuring the induction of the adipocyte-differentiation markers adiponectin (Adipoq), CD36 and PPARγ by real-time PCR (compare Fig. 1B). All three markers were up regulated in both cell passages upon differentiation with highest expression levels between day 7 and 10 after differentiation. In parallel, adipocyte-differentiation could be detected by visual inspection of the cell cultures. While undifferentiated cells showed a flat and fibroblast-like shape (Fig. 1C), cells became more and more rounded during the differentiation process. In parallel, the formation of multilocular lipid droplets in the cytoplasm could be observed in differentiating adipocytes which became bigger during this process (compare Fig. 1D). These vesicles could specifically be stained with Oil Red O which was a further proof of the successful adipocyte differentiation (data not shown). Cells from both passages showed slightly different kinetics of adipogenesis; while cells from passage 5 were terminally differentiated at day 7 after induction, cells from passage 8 differentiated slightly slower and reached this stage at day 10 after initiation of adipocyte differentiation. This was reflected both by the expression pattern of adipocyte marker genes as well as by the morphology of the cells. Because of this obvious difference between both cell passages, we included day 10 of passage 8 in the following analyses whereas for passage 5, day 7 was the last time point that was included in the following miRNA expression profiling.

### Screening of miRNA expression in MSC-derived differentiated adipocytes

**Figure 2:**
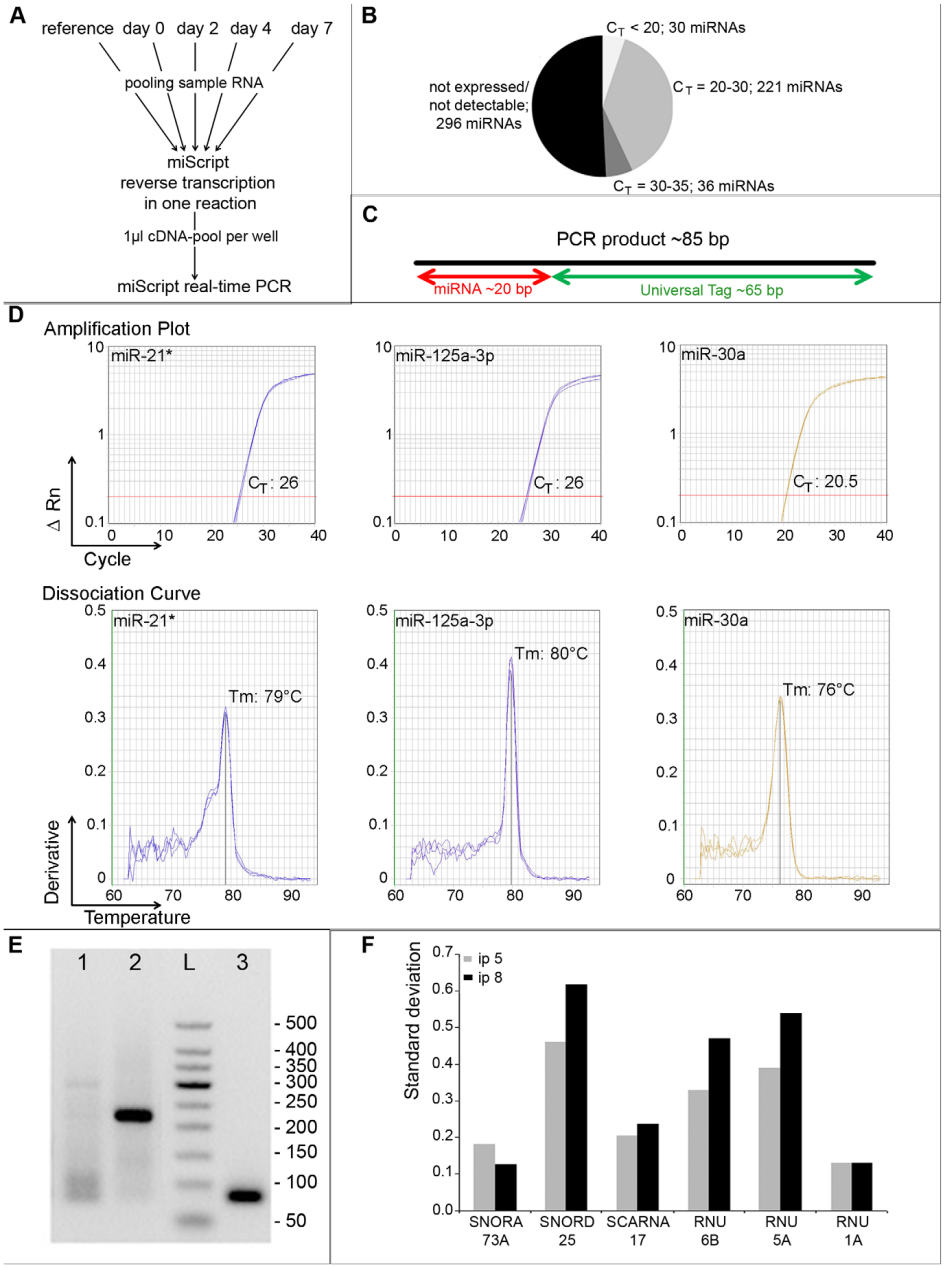
Real-time PCR based profiling of miRNAs being differentially regulated during the course of MSC differentiation to adipocytes. **A**: RT-PCR based pooling strategy to identify all miRNAs being expressed in any of the samples taken during the differentiation time-course. **B**: Summary of the expression levels of 583 miRNAs analyzed with the pooling strategy. **C**: Schematic overview of a typical miRNA PCR amplicon produced by SYBR Green based miScript PCR assays. PCR products are typically around 85 bp long and of these 20 bp resemble the variable miRNA sequence while the remaining 60 bases contain the universal PCR primer binding site introduced during reverse transcription. **D–E**: Melting curve analysis (**D**) in combination with agarose gel electrophoresis (**E**), Lane 1: miR-21*, lane 2: miR-125a-3p, lane 3: miR-30a PCR-products, “L”: GelPilot 50 bp Ladder revealed the specificity of miRNA PCR products. **F**: Identification of the most suitable housekeeping gene for the normalization of the miRNA expression data. For this purpose the expression of six small RNAs was analyzed at all time points and in both cell passages. To identify the small RNA showing the lowest variation over the whole sample set the mean values and standard deviations of all time points were calculated for every gene and cell passage. The table shows the standard deviations of these means.

A PCR-based approach employing miRNA-specific primers for all known mouse microRNAs (583 in miRBase v.12) was used to profile the expression of these molecules during adipogenesis. In an initial screening, we used pools of five RNA samples from passage 5 to get first information about the overall expression of the complete miRNome that could be detected in these cells. For this purpose, we pooled RNA from undifferentiated proliferating MSC (as reference), and from cells of days 0, 2, 4 and 7 after initiation of adipogenesis. This procedure (as depicted in Fig. 2A) allowed us to quickly sort out miRNAs not being expressed in any of the samples irrespective of their differentiation status. By using this pooling approach, we found that 296 of all miRNAs were absent in the pooled samples (C_T_s of >35), and that 36 miRNAs were only weakly expressed (C_T_ values between 30 and 35). 221 miRNAs showed an average expression level (C_T_s between 20 and 30) and 30 miRNAs displayed high expression levels with C_T_ values less than 20 (Fig. 2B). The specificity of the amplified PCR-products was assessed by melting curve analysis. Because of the largely uniform nature of the miRNA-specific PCR amplicons (compare Fig. 2C), the expected Tm for true positive PCR products should be 75°C (+/− 2°C). The specificity of most miRNA PCR assays leading to detectable C_T_ values could therefore be confirmed by analyzing their respective melting curves. As shown in Fig. 2D and 2E, specific PCR products with melting temperatures of about 75°C did also posses the expected size of 85 bp demonstrated by agarose gel electrophoresis (a representative result for mir-30a is given). Thus, unspecific PCR products displaying high Tm values could be easily identified by melting curve analysis. Melting temperatures of about 80°C or higher indicate that the PCR product is most likely unspecific. In this rare event, subsequent gel analysis revealed single or multiple PCR products of the wrong size. The two examples shown here are miR-21* displaying a melting curve peak at 79°C and multiple PCR products ranging in size between 80 and 300 bp and miR-125a-3p exhibiting a Tm of 80°C and one distinct PCR product of about 230 bp (see Fig. 2D and 2E). Such microRNA PCR assays giving rise to PCR products not passing the quality criteria described here were subsequently excluded form further analysis.

For appropriate normalization of the miRNA expression values, we intended to identify an endogenous small housekeeper transcript showing low expression variation during adipogenesis. For this purpose, we separately analyzed the expression of the six small RNAs SNORA73A, SNORD25, SCARNA17, RNU6B, RNU5A and RNU1A in all samples taken during the differentiation of both MSC passages 5 and 8. These small transcripts are highly suitable candidate RNAs for normalization of miRNA expression levels because they are ubiquitously expressed, have small sequence lengths of 45–200 bp and are, like mature miRNAs, not polyadenylated. The arithmetic mean C_T_ and standard deviation of all time-course samples were determined for both cell passages (Fig. 2F shows the standard deviation of the C_T_ -values as a measure of the expression variation during the differentiation time course). The small non-coding RNAs showing the smallest variation of expression in both cell passages were snoRNA73A and RNU1A. We selected SNORA73A exhibiting a standard deviation of 0.12 and 0.18 respectively and not RNU1A because the latter displayed less consistent amplification plots (data not shown).

**Figure 3:**
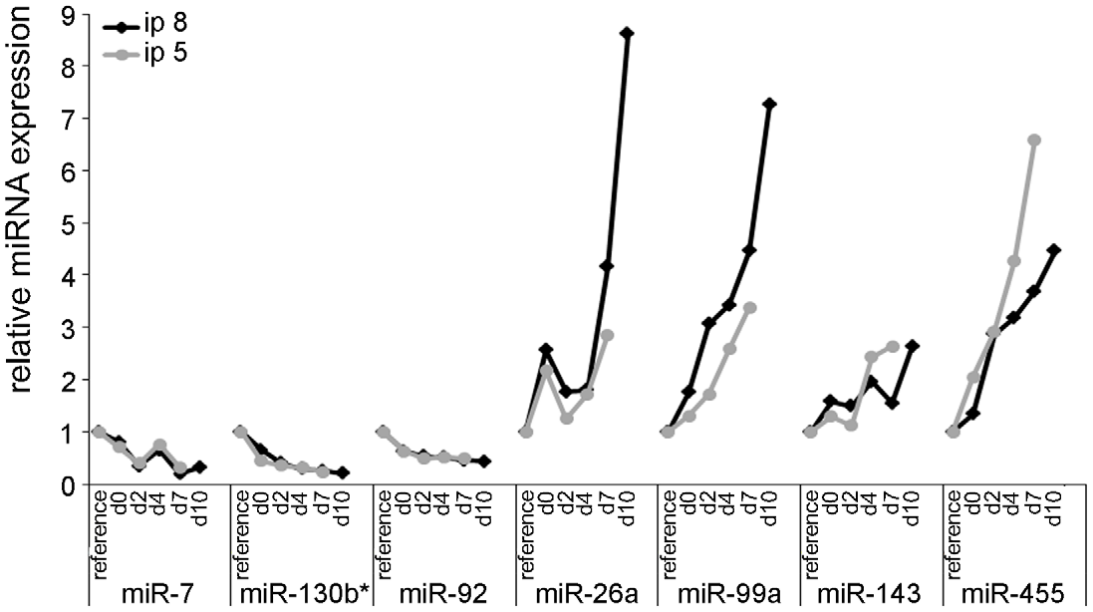
Expression kinetics of miRNAs being differentially regulated during differentiation of MSC to adipocytes. The expression of miRNAs was analyzed by real-time PCR individually at all time points during the differentiation course and for two independent cell passages (5 and 8) to identify miRNAs being regulated during adipogenesis of murine mesenchymal stem cells. Data were normalized to SNORA73A and plotted as relative miRNA expression levels compared to the level of undifferentiated subconfluent reference cells.

The expression patterns of the 287 miRNAs, that were detectably expressed in the pooled samples, were reanalyzed in both cell passages and for each individual sample of the MSC differentiation time course. We found a total number of 66 miRNAs showing expression changes comparing undifferentiated dividing cells with differentiated adipocytes in both cell replicates (including days d0, d2, d4, and d7 in the case of passage 5 and d0, d2, d4, d7 and d10 for passage 8) with fold changes higher than 2 or less than 0.5. The individual expression data for each of the 66 differentially regulated miRNAs is listed in supporting table S1. Of these, 56 miRNAs were upregulated during adipogenesis whereas ten of them showed reduced expression in differentiated cells. Members of 5 miRNA families were coordinately up regulated during adipogenesis, namely let-7a, let-7c, let7-e, let-7g, and let-7i; members of the miR-29 family miR-29a, miR-29b, and miR-29c; miR-30a_1, miR-30b, miR-30c, miR-30d, and miR-30e; miR-181a, and miR-181b; miR-199a, miR-199a-3p, miR199-b*. Besides these, miR103/107 and miR-143, were also found to be upregulated in the adipogenesis model analyzed here. Fig. 3 is showing the representative expression kinetics for four upregulated and three down regulated miRNAs in both cell passages.

### Analysis of miRNAs regulated early after induction of adipogenesis

**Figure 4:**
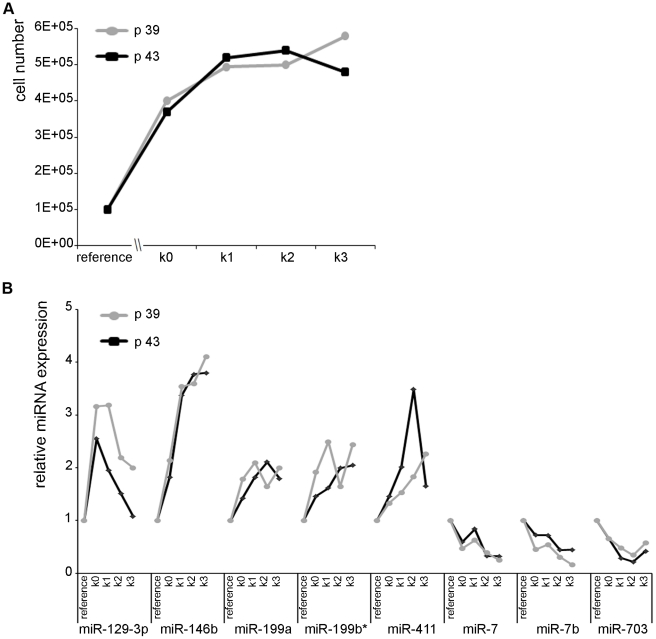
Analysis of growth arrested NIH/3T3-cells to identify miRNAs regulated due to quiescence of cells. **A**: Cell numbers of two passages of NIH/3T3-cells were counted at subconfluence, at the day of confluency and at the following three days to identify the time-point of complete proliferation arrest. **B**: Expression kinetics of miRNAs during proliferation arrest of NIH/3T3 cells were determined by RT-PCR. Data were normalized to SNORA73A and plotted as relative expression changes compared to subconfluent reference cells.

The differentiation of mesenchymal stem cells into adipocytes requires two steps, cell cycle arrest and treatment with differentiating agents (IBMX, DMX). To identify the miRNAs that have been up- or down-regulated because of the growth arrest of the MSCs, confluent non-dividing NIH/3T3 mouse fibroblasts, which are not capable of differentiating into adipocytes, were tested as well. Cell counting of two individual cell passages (passage 39 and passage 43) revealed that cells stopped dividing one day after reaching confluency (Fig. 4A). The expression of the 66 microRNAs being regulated during adipogenesis of mesenchymal stem cells was also determined from growth arrested NIH/3T3 cells at four different time points (k_0_–k_3_) and compared to a reference of subconfluent dividing NIH/3T3 cells. In MSCs, some miRNAs were differentially regulated in both cell passages early after IBMX, DMX treatment with fold-changes >2 at day 2 after induction (e.g. miR-455; Fig. 3). In the confluent NIH/3T3 cells, only a small number of miRNAs was up- or down-regulated more than two-fold, five miRNAs showed induction of expression in both passages, three miRNAs were down-regulated at confluency (Fig. 4B). Five of these eight miRNAs (miR-199a, miR-7, miR-146b, miR-7b, and miR-199b*) were similarly regulated early during the adipogenesis process of MSCs, indicating that their regulation was due to cell cycle arrest rather than adipogenesis. Whereas miR-411 and miR-703 were regulated late during the adipocyte differentiation process and miR-129-3p was up-regulated in growth-arrested NIH/3T3 cells but down-regulated in MSCs, indicating that these three miRNAs play different roles in NIH/3T3 cells and differentiating MSCs.

### *In vivo* miRNA expression in adipose tissue from obese mice

**Figure 5:**
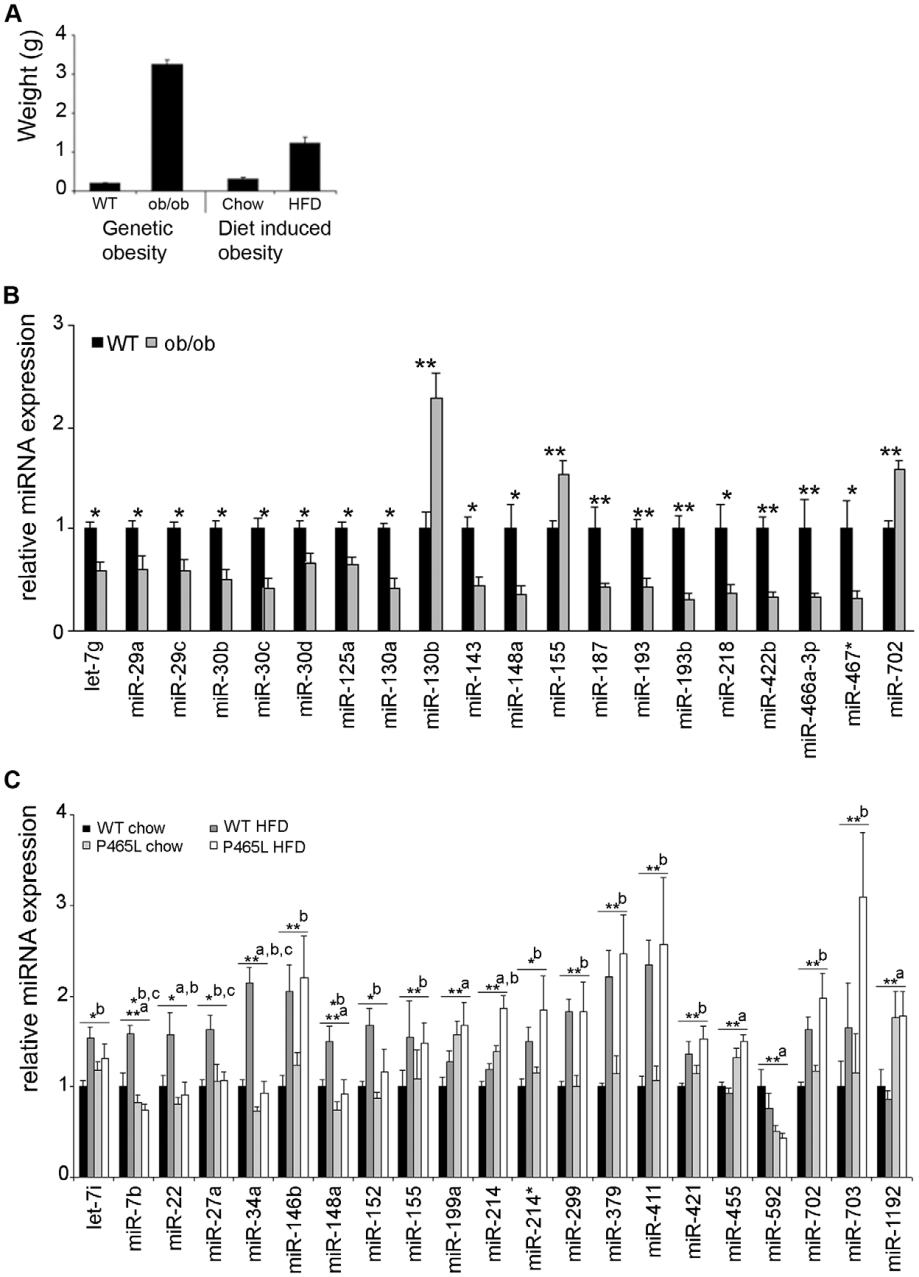
Differential expression of miRNAs in epididymal fat pads from mouse models of genetically or diet induced obesity. **A**: Epididymal fat pad weights were compared of obese ob/ob mice and wild-type controls (Ob/ob model) and of wt or PPARγ DN mice fed either a chow or a high-fat diet (DIO model). **B**: miRNA levels were analyzed in epididymal fat pads from five wild-type (WT) and five leptin-deficient ob/ob mice by real-time-PCR. Data were normalized to SNORA73A expression. The relative expression levels compared to wild-type mice were plotted and significance was calculated using student's t-test. * p<0.05 ; **p<0.01 **C**: For the diet induced obesity model, miRNA expression in epididymal fat pads from chow or high fat diet fed wild-type as well as from PPARγ DN (P465L) mice was determined by real-time PCR. Five mice were analyzed for each condition. Data were normalized to SNORA73A expression and plotted as relative expression levels compared to wild-type normally fed mice. Anova tests revealed significantly regulated miRNAs * p<0.05 ; **p<0.01 and influence of genome (a), diet (b) or interaction of both (c).

To gain further insight into the role of the microRNAs that have been found to be differentially expressed during adipocyte differentiation of mesenchymal stem cells, we investigated the transcription patterns of these miRNAs in white adipose tissue derived from several *in vivo* model systems. As a model for genetic obesity we used leptin-deficient ob/ob mice that were compared with corresponding wt littermates. In the second model, obesity of the animals was induced by high fat diet (as described in materials and methods) and these mice were compared with littermates fed a chow diet. The degree of obesity is displayed by the epididymal fat pad weight of the corresponding animals (Fig. 5A). In ob/ob-mice, the epididymal fat pad weight was 3.24 g, more than 15 fold greater than that of the corresponding control animals (n = 8 per group). In the case of the diet induced obesity model, the fat pad weight of HFD animals was 1.24 g, 4 fold greater than that of the control group (n = 6 per group). By comparing white fat tissue of leptin-deficient ob/ob mice with the corresponding tissue from wild type mice with the same genetic background (C57BL/6), we found 20 miRNAs being differentially expressed (cutoff: 1.5-fold up regulation and 0.66 fold down regulation; p<0.05). Of these 20 miRNAs three were up-regulated in ob/ob-mice whereas the other 17 were down-regulated compared to wt animals. Student's t-test revealed significant changes (p<0.05) for 12 of the regulated miRNAs and highly significant changes (p<0.01) for 8 miRNAs (Fig. 5B). Interestingly, we found that the majority of those miRNAs (17/20) that were upregulated in differentiating adipocytes were downregulated in the fat pads of obese mice and vice versa. Only three miRNAs were consistently regulated in both model systems with miR-155 being upregulated both in differentiating adipocytes and in obese mice and miR-466a-3p as well as miR-467 that were downregulated in both adipocytes and obese mice.

Additionally, we investigated the influence of high fat diet and PPARγ activity on microRNA expression either separately or in combination. For this purpose, animals with a mixed genetic background harboring either the PPARγ wild type gene (WT) or a dominant-negative PPARγ mutation (P465L DN) were fed either a high fat diet or a chow diet. The levels of the 66 miRNAs being differentially expressed during in vitro adipocyte development, were also determined in epididymal fat pads of the four *in vivo* conditions (wt+chow, wt+HFD, P465L+chow, P465L+HFD). Compared to wt chow fed animals that served as a reference 21 miRNAs were differentially expressed in at least one of the other three conditions (fold change >1.5 or <0.66; p<0.05). Using 2-way ANOVA, the influence of the genotype (a), diet (b) or an interaction of both factors (c) on the miRNA expression was assessed (compare Fig. 5C). For eleven of these regulated miRNAs, the diet was the only factor responsible for altered expression levels (“b” in Fig. 5C). In all these cases, the miRNA expression was induced after high fat diet in both the wild-type as well as PPARγ mutant animals. The high fat diet induced up-regulation of miR-421, miR-702 and miR-703, in these mouse models was not reflected in the mesenchymal stem cell model of adipogenesis as these miRNAs were down-regulated during *in vitro* adipocyte differentiation. The expression of miR-146b that was induced both early after induction of mesenchymal stem cell differentiation as well as after reaching a growth arrest in NIH/3T3 fibroblasts, was also significantly up-regulated upon HFD-treatment in wild-type mice as well as mutant animals. The expression of four miRNAs was only dependent on the PPARγ expression status of the animals; miR-199, miR-455 and miR-1192 were upregulated in mice with dominant-negative PPARγ irrespective of the diet given to the animals, the fourth, miR-592, was reduced in mutant animals (“a” in Fig. 5C). An interaction between genotype and diet on miRNA expression was found in three cases: miR-7b, miR-27a and miR-34a were up-regulated in wt animals receiving high fat diet but not upregulated in P465L DN mice receiving HFD (“c” in Fig. 5C).

## Discussion

### Experimental strategy for miRNA profiling

In the present study, we have analyzed the expression pattern of 583 mouse miRNAs during the adipogenic differentiation of murine mesenchymal stem cells. The expression profile has been performed by qRT-PCR employing a pooling strategy. Using this new approach, we were able to analyze the expression of every single mouse miRNA (miRBase v.12) at different time points during the differentiation process of murine mesenchymal stem cells into terminally differentiated adipocytes. The pooling of samples significantly reduces work load while the sensitivity of a real-time PCR based approach significantly exceeds other alternative methods like Northern blot or even miRNA expression microarrays. Using such a pooling approach we showed that complex PCR-based miRNA screening projects could be handled in a reasonable time, even by laboratories devoid of extensive lab automation. This strategy could also be employed as a primary screen profiling microRNA expression in different animal tissues (e.g samples from a variety of brain regions). After the identification of miRNAs that are expressed in any of the pooled samples, the tissue-specific expression of these “primary hits” can be determined by qRT-PCR analysis of the individual samples. In such a study design, the primary screening serves only for the qualitative detection, whereas for the quantitative analysis of microRNA expression, each sample needs to be tested individually.

Using pooled samples results in the dilution of each individual sample within the pool, with the consequence of reduced sensitivity during quantitative PCR analysis. The theoretical loss of sensitivity can be calculated as follows: given an ideal amplification efficiency of 100% (E = 100%) with a doubling of the PCR product with each amplification cycle, the slope of a dilution curve (S) could be calculated as −3.322 using the following equation: E = (10e(−1/S)−1)×100. This means that a ten-fold reduction of template-DNA would result in a 3.322 higher C_T_ value. By pooling five cDNA samples, as we did in the present study, the copy number for a specific target cDNA from one sample is diluted 5-times in the resulting pooled cDNA. Therefore, the loss of sensitivity for each individual sample in the pool is small, as the C_T_ value of transcripts present in only one of the five pooled samples would be 2.322 C_T_ higher than in the individually analyzed sample. Statistically, an average C_T_ value of 35 means, that there are about five copies of the miRNA transcript detected in the PCR reaction [20]. That is why there is no biological relevance of transcripts detected with C_T_ values >35. According to that, the limit of detection in pools of 5 samples is lowered to a C_T_ of 32.7.

A big advantage of the SYBR Green-based miRNA PCR assays used in this study is the possibility to quickly verify the specificity of the amplified sequences by studying the melting curves of the miRNA-specific PCR products. These largely uniform amplicons should all display very similar Tms of 75°C. In the rare case that PCR-products with significantly higher melting temperatures were produced these have been proven to be not specific using agarose gel-based fragment size determination. There may be a theoretical risc for cross reactivity between closely related microRNA isoforms, such as from the let-7 family, not distinguishable by melting curve analysis alone. Nevertheless, a recent study has provided evidence for the relatively high specificity of the miRNA PCR assays employed in our present study [21]. For several members of the let-7 family, Kumar et al. could demonstrate that the amplification of related miRNAs was much less efficient than that of the actually analyzed miRNA. Therefore, these unspecific amplicons contribute only to a lesser part to the overall signal.

Technically, the pooling of samples for RT-PCR analysis -besides economical aspects- has several benefits compared to gene chip technology platforms usually employed for such screening approaches. Since microarray platforms are less sensitive in the detection of lowly expressed genes [20], pooling of several RNA preparations is not recommended. In direct comparison, the sensitivity of a PCR-based detection with a “Limit of Detection” of ten copies is 1000-times higher than what could be achieved with microarrays [20] [22]. The specificity of the hits of such a microarray analysis could not be easily verified. This in particular is an important aspect to consider when thinking of the detection limitations introduced by the short length of the target structures (mature miRNAs with a length of 21–23 base-pairs). In contrast to current mRNA expression arrays, where multiple target-specific probe sets per gene are employed to guarantee specificity, such a redundant design is not feasible for miRNA microarrays.

### miRNA expression profiling of mesenchymal stem cell derived adipocytes

The murine bone-marrow-derived mesenchymal stem cells used in this study have the potential to differentiate into adipocytes. Accumulation of lipid droplets in the cytoplasm of differentiating cells could be easily used to estimate the percentage of fat cells in the culture. Such stem cell derived adipocytes are a relevant model for white fat tissue differentiation and may help to better understand the underlying differentiation mechanisms and to find potential therapeutic targets for obesity or metabolic diseases.

Among the 66 miRNAs found in our study to be differentially regulated in MSC-derived adipocytes, miR-103/107 and miR-143 have already been found to play a crucial role in adipocyte differentiation [23], [24]. The direct involvement of miR-143 in adipocyte differentiation has been studied by using antisense oligonucleotides in cultured human pre-adipocytes. In this model, blocking of miR-143 reduced the expression of adipocyte differentiation markers by 50% [25]. Opposite effects have been observed after ectopical expression of miR-143 in 3T3-L1 cells which led to the up-regulation of adipocyte marker genes [24]. It has also been reported that elevated expression levels of miR-103 accelerate adipocyte differentiation upon treatment with differentiation factors [24].

The terminal differentiation of mesenchymal stem cells into mature adipocytes *in vitro* is a process involving two major steps: firstly, cells need to be growth arrested, and secondly the treatment with differentiating agents induces the transformation into adipocytes (reviewed in [17]). In order to be able to distinguish miRNA expression changes that were rather related to adipocyte differentiation from those that occurred as a result of growth arrest alone, we analyzed the expression of the 66 candidate miRNAs in growth arrested mouse fibroblast cell line NIH/3T3, which does not have the potential to differentiate into adipocytes. Comparing growth arrested cells to normally dividing control cells, the expression of 8 of the 66 analyzed miRNAs was affected by growth arrest in NIH/3T3 cells (fold-changes >+/−2). Among those, were miR-7a, miR-7b, miR-146b, miR-199a and miR-199b* that were regulated at very early time-points after induction of MSCs and show the same direction of up- or down-regulation in the NIH/3T3 model. Comparing our data with those obtained with the 3T3-L1 adipogenesis model used by Xie and coworkers further supports the assumption, that these miRNAs may not have a direct role in the adipogenic differentiation process [24]. Only miR-146b is found to be moderately up-regulated, and none of the other seven miRNAs is significantly changed in this study. A direct involvement of miR-146b in adipogenesis has not yet been described to our knowledge. Recently it has been shown that miR-146b expression could suppress breast cancer metastasis [26], supporting our finding of its involvement in growth regulation rather than adipogenesis.

Besides the regulation of miR-103/107 and miR-143, we could also confirm the up-regulation of miR-422b, miR-148a, miR-30c, and miR30a-5p found in differentiating 3T3-L1 cells by Xie and colleagues [24]. Nine of the miRNAs we found to be regulated have also been described by Esau and co-workers in human white adipocytes [25]. Although differentiated adipocytes have been compared to pre-adipocytes in this study, the overlap was high. Besides miR-103/107 and miR-143, miR-30c was also upregulated. These three miRNAs were the only ones that were consistently upregulated in our analysis as well as in the work of Esau et al. (2004) as well as in Xie et al. (2009). In general, the number of significantly regulated miRNAs we found differentially expressed was higher compared to the other two studies [24]. One obvious explanation is the number of miRNAs that were investigated in the individual studies. Esau and coworkers analyzed differential expression of 254 human and mouse miRNAs and Xie et al. (2009) used gene chip arrays based on miRBase version 9 for the analysis of 375 miRNAs in differentiating 3T3-L1 cells, We have anlaysed the expression of all mouse miRNAs published in miRBase 12, a total of 583 miRNA sequences.

### miRNA expression in adipose tissue from obese mice

Studying the miRNA regulation in different *in vivo* models can help to strengthen the biological relevance of the findings received from the *in vitro* differentiation model employed here. In the present study, we have used ob/ob mice as a genetic model for obesity. As a second model, we analyzed fat tissue from mice with diet-induced obesity. either having a normal genetic background or harboring a dominant negative mutation of PPARγ.

Comparing white fat tissue from obese leptin-deficient ob/ob-mice with tissue from normally fed wild type mice revealed that most of the upregulated miRNAs in the differentiated MSC model were downregulated in fat pads from these obese mice and vice versa. Only a small number (3/20) of microRNAs were consistently regulated in both model systems. Similar observations have been made earlier for adipogenic genes that are normally up-regulated during adipogenesis, but show reduced expression in epididymal fat pads of ob/ob mice [27]–[29]. The reverse regulation of miRNA expression in white fat tissue of ob/ob-mice compared to differentiated 3T3-L1 adipocytes has also been reported earlier [24]. In this study, miRNAs being regulated in white fat of ob/ob-mice are similarly regulated in TNFα treated differentiated 3T3-L1 adipocytes. As TNFα treatment may induce insulin resistance by reactivating the expression of preadipocyte genes *in vitro* [30] [24] this may be the reason why the majority of miRNAs found in our study are oppositely regulated in differentiated, insulin-sensitive adipocytes and insulin resistant ob/ob mice. Alternatively, mature adipose tissue is quite different in terms of its make up to a simple adipocyte culture model. Adipose tissue contains multiple cell types including adipocytes, preadipocytes, stem cells, endothelial cells, macrophages and multiple other immune cells. The differential regulation we observed between the MSC culture system and the effect of obesity on adipose tissue in vivo may reflect the regulation of miRNAs in non-adipocyte or preadipocyte cell types.

Using our second mouse model of obesity, the influence of a high fat diet on miRNA expression could be analyzed either physiologically or under conditions when PPARγ activity was reduced. By comparing lean PPARγ wt animals on a chow diet with obese littermates receiving a high fat diet, normally fed PPARγ DN and PPARγ DN mice with diet-induced obesity, we were able to differentiate effects only induced by diet from those effects observed as a consequence of reduced PPARγ activity and effects due to an interaction between diet and genotype. Interestingly, only four of the 21 regulated miRNAs (miR-199a, miR-455, miR-592, and miR-1192) were solely dependent on the genotype; independent of diet, the expression of miR-199a, miR-455 and miR-1192 was up-regulated in PPARγ DN animals whereas miR-592 was down-regulated in these mice compared to wt. After heterodimerization with the retinoic acid-like receptor (RXR), PPARγ recognizes the peroxisome proliferator response element (PPRE), that is located in the promoters of many adipocyte-specific target genes and thereby acts as transcription activator recruiting other transactivators like PGC-1 [31]. Therefore the diet-independent downregulation of miR-592 in PPARγ DN animals indicates that miR-592 expression is directly or indirectly (if e.g. co-expressed as an intronic miRNA within the transcript of a PPARγ-responsive host gene) driven by PPARγ. As miR-199a, miR-455 and miR-1192 were up-regulated in PPARγ DN animals this may indicate that PPARγ normally inhibits their expression directly (e.g. by recruiting transcriptional inhibitors to those promoters) or indirectly (by the PPARγ specific transctivation of a transcriptional inhibitor).

Eleven of the 21 miRNAs were found to be up-regulated in these animals upon feeding a high fat diet, irrespective of the PPARγ activity. Among those, let-7i, miR-146b, miR-152, miR-155, miR-214*, miR-299 and miR-411 are similarly regulated in differentiating MSCs, whereas miR-421, miR-702 and miR-703 were oppositely regulated in MSCs. According to our statistical analysis, the regulation of miR-7b, miR27a and miR-34a is influenced by diet and genotype of the animals together. In PPARγ-wt animals, administration of a high fat diet leads to significantly higher expression levels compared to lean control animals, whereas PPARγ-dominant negative animals do not show increased expression levels of these microRNAs when receiving the same high fat diet. This indicated that PPARγ activity is indispensable for the HFD induced expression of these three miRNAs. In a recent study investigating the influence of the body mass index (BMI) of obese and lean human donors on miRNA expression in the subcutaneous fat depot, miR-34a was found to positively correlate with BMI >30.0 Kg/m^2^ [32]. On the other hand the expression levels of miR-34a remains constant in our leptin-deficient ob/ob mice as well as PPARγ-dominant negative HFD animals in comparison to the respective control group even though the degree of obesity in these animals is at least comparable to or in the case of ob/ob mice much higher than in the group of PPARγ-wt HFD mice. These findings suggest that miR-34a is only up-regulated in obese individuals having a normal genetic background and with normal levels of PPARγ activity.

Overall there were relatively few miRNAs that were similarly regulated in the ob/ob model and by HFD. This may be because of the differing degrees of obesity or due to a specific effect of the lack of leptin in the ob/ob mouse. However, it is important to note that the dietary-induced obesity model and the ob/ob mouse model were on different genetic backgrounds (c57/bl6 for the ob/ob, a mixed C57/bl6/sv129 background for the DIO model). Genetic background can have significant effects in terms of the response of mice to HFD [33] and it is possible the different patterns between DIO and genetic obesity were driven by the different genetic backgrounds. However, the fact miR702 and miR155 were co-ordinately regulated in the DIO and genetic obesity models, despite the differences in genetic background, provides additional robust evidence for the generality of these changes in adipose tissue in response to obesity.

We studied the global expression pattern of all known murine miRNAs during the differentiation process of mesenchymal stem cells into terminally differentiated adipocytes. In this model system, we identified a collection of 66 miRNAs with significantly altered expression. Comparing our findings with similar experimental approaches we could confirm the involvement of certain miRNAs in the adipogenic differentiation process. Some other miRNAs have not yet been described before in this context. Further studies will unveil the relevance of these newly discovered miRNAs with regard to their suitability to serve as biomarkers or even therapeutic targets for obesity and obesity-related diseases.

## Supporting Information

Table S1 **Expression data for 66 miRNAs, differentially regulated during adipogenesis of multipotent adult mouse mesenchymal stem cells.**
(DOC)
