# Supplementary material for: Genome-Wide Profiling of MicroRNAs in Adipose Mesenchymal Stem Cell Differentiation and Mouse Models of Obesity
Source: PLoS One. 2011 Jun 23;6(6):e21305. doi: 10.1371/journal.pone.0021305 (PMC3121761; doi:10.1371/journal.pone.0021305)
Supplement: Table S1 — Expression data for 66 miRNAs, differentially regulated during adipogenesis of multipotent adult mouse mesenchymal stem cells. (DOC) [file pone.0021305.s001.doc]

**Table S1:**

| **Primer** | **Passage** | **Routine** | **Day 0** | **Day 2** | **Day 4** | **Day 7** | **Day10** |
| --- | --- | --- | --- | --- | --- | --- | --- |
| Mm_let-7a | 8 | 1 | 1,26 | 1,37 | 1,57 | 1,71 | 2,60 |
|  | 5 | 1 | 2,02 | 2,22 | 2,08 | 2,85 |  |
| Mm_let-7c | 8 | 1 | 1,28 | 1,62 | 1,60 | 1,74 | 2,32 |
|  | 5 | 1 | 1,80 | 2,09 | 2,05 | 2,90 |  |
| Mm_let-7e | 8 | 1 | 1,29 | 1,29 | 1,45 | 1,90 | 2,45 |
|  | 5 | 1 | 1,81 | 1,52 | 1,29 | 2,11 |  |
| Mm_let-7i | 8 | 1 | 2,04 | 1,92 | 1,71 | 1,52 | 2,64 |
|  | 5 | 1 | 1,94 | 2,36 | 2,99 | 2,53 |  |
| Mm_let-7g | 8 | 1 | 2,07 | 2,13 | 2,35 | 1,92 | 3,40 |
|  | 5 | 1 | 1,60 | 1,97 | 2,19 | 2,38 |  |
| Mm_miR-7 | 8 | -1 | -1,24 | -2,89 | -1,54 | -5,06 | -3,15 |
|  | 5 | -1 | -1,42 | -2,53 | -1,31 | -3,05 |  |
| Mm_miR-7b | 8 | -1 | -1,38 | -3,36 | -2,07 | -8,50 | -3,40 |
|  | 5 | -1 | -1,74 | -5,54 | -2,06 | -5,07 |  |
| Mm_miR-16 | 8 | 1 | 1,39 | 1,79 | 1,87 | 1,82 | 2,52 |
|  | 5 | 1 | 1,70 | 1,71 | 2,49 | 2,50 |  |
| Mm_miR-22 | 8 | 1 | 1,69 | 1,54 | 1,82 | 2,00 | 2,48 |
|  | 5 | 1 | 1,18 | 1,27 | 1,82 | 2,67 |  |
| Mm_miR-24 | 8 | 1 | 1,38 | 1,55 | 1,56 | 1,78 | 2,79 |
|  | 5 | 1 | 1,64 | 1,78 | 1,87 | 3,18 |  |
| Mm_miR-26a | 8 | 1 | 2,56 | 1,76 | 1,79 | 4,16 | 8,61 |
|  | 5 | 1 | 2,17 | 1,25 | 1,72 | 2,86 |  |
| Mm_miR-27a | 8 | 1 | 1,35 | 1,23 | 1,71 | 1,49 | 2,75 |
|  | 5 | 1 | 1,15 | 1,33 | 1,39 | 2,57 |  |
| Mm_miR-29a | 8 | 1 | 1,46 | 2,22 | 2,32 | 2,26 | 3,46 |
|  | 5 | 1 | 1,44 | 1,88 | 2,70 | 3,00 |  |
| Mm_miR-29b | 8 | 1 | 1,86 | 2,89 | 3,22 | 2,66 | 5,01 |
|  | 5 | 1 | 1,50 | 1,86 | 3,32 | 4,21 |  |
| Mm_miR-29c | 8 | 1 | 1,44 | 2,12 | 2,04 | 2,22 | 3,05 |
|  | 5 | 1 | 1,37 | 1,90 | 2,17 | 3,05 |  |
| Mm_miR-30a | 8 | 1 | 1,56 | 1,80 | 2,07 | 2,76 | 3,67 |
|  | 5 | 1 | 1,55 | 1,52 | 1,71 | 3,51 |  |
| Mm_miR-30b | 8 | 1 | 2,52 | 2,82 | 3,36 | 3,70 | 7,19 |
|  | 5 | 1 | 1,48 | 1,57 | 2,26 | 4,17 |  |
| Mm_miR-30c | 8 | 1 | 2,19 | 2,73 | 3,03 | 3,99 | 6,03 |
|  | 5 | 1 | 1,34 | 1,63 | 2,53 | 4,14 |  |
| Mm_miR-30d | 8 | 1 | 1,51 | 1,75 | 1,97 | 2,61 | 3,14 |
|  | 5 | 1 | 1,67 | 1,39 | 2,04 | 3,51 |  |
| Mm_miR-30e | 8 | 1 | 1,53 | 1,43 | 1,51 | 1,93 | 2,83 |
|  | 5 | 1 | 1,66 | 1,19 | 1,76 | 2,91 |  |
| Mm_miR-34a | 8 | 1 | 1,85 | 1,86 | 2,57 | 2,02 | 3,70 |
|  | 5 | 1 | 1,54 | 1,71 | 2,55 | 3,89 |  |
| Mm_miR-92 | 8 | -1 | -1,59 | -1,89 | -1,98 | -2,20 | -2,32 |
|  | 5 | -1 | -1,59 | -2,07 | -1,91 | -2,04 |  |
| Mm_miR-99a | 8 | 1 | 1,75 | 3,07 | 3,42 | 4,46 | 7,26 |
|  | 5 | 1 | 1,31 | 1,72 | 2,58 | 3,38 |  |
| Mm_miR-101a | 8 | 1 | 3,35 | 3,91 | 4,76 | 5,15 | 10,85 |
|  | 5 | 1 | 1,70 | 1,31 | 2,40 | 4,56 |  |
| Mm_miR-101b | 8 | 1 | 1,44 | 2,43 | 2,91 | 1,56 | 3,32 |
|  | 5 | 1 | 1,96 | 2,37 | 3,30 | 3,91 |  |
| Mm_miR-103 | 8 | 1 | 2,18 | 1,84 | 2,06 | 1,91 | 3,16 |
|  | 5 | 1 | 1,53 | 1,80 | 2,44 | 2,57 |  |
| Mm_miR-125a | 8 | 1 | 1,22 | 1,69 | 1,55 | 1,83 | 2,48 |
|  | 5 | 1 | 1,09 | 1,48 | 2,17 | 2,10 |  |
| Mm_miR-129-3p | 8 | -1 | -2,81 | -2,36 | -2,80 | -3,37 | -4,03 |
|  | 5 | -1 | -2,82 | -2,17 | -1,78 | -2,38 |  |
| Mm_miR-130a | 8 | 1 | 1,95 | 1,71 | 1,61 | 1,84 | 2,58 |
|  | 5 | 1 | 1,33 | 1,32 | 2,32 | 2,66 |  |
| Mm_miR-130b* | 8 | -1 | -1,53 | -2,52 | -3,49 | -3,96 | -4,88 |
|  | 5 | -1 | -2,23 | -2,79 | -3,21 | -4,23 |  |
| Mm_miR-143 | 8 | 1 | 1,57 | 1,49 | 1,96 | 1,55 | 2,64 |
|  | 5 | 1 | 1,29 | 1,12 | 2,43 | 2,64 |  |
| Mm_miR-146 | 8 | 1 | 6,17 | 5,08 | 5,32 | 4,76 | 5,25 |
|  | 5 | 1 | 2,48 | 1,64 | -1,20 | 1,09 |  |
| Mm_miR-146b | 8 | 1 | 9,13 | 6,37 | 4,11 | 2,38 | 2,22 |
|  | 5 | 1 | 4,18 | 2,79 | 2,56 | 1,31 |  |
| Mm_miR-148a | 8 | 1 | 4,96 | 4,00 | 5,69 | 5,03 | 9,84 |
|  | 5 | 1 | 2,81 | 2,63 | 5,92 | 6,48 |  |
| Mm_miR-152 | 8 | 1 | 2,27 | 1,83 | 2,26 | 1,90 | 2,76 |
|  | 5 | 1 | 1,41 | 1,29 | 2,03 | 2,14 |  |
| Mm_miR-155 | 8 | 1 | 3,16 | 3,93 | 3,03 | 2,11 | 2,13 |
|  | 5 | 1 | 2,09 | 2,85 | 2,43 | 1,95 |  |
| Mm_miR-181a | 8 | 1 | 1,29 | 2,03 | 1,91 | 3,29 | 3,89 |
|  | 5 | 1 | 1,37 | 2,12 | 1,79 | 3,89 |  |
| Mm_miR-181b | 8 | 1 | 1,36 | 1,82 | 2,04 | 2,20 | 3,74 |
|  | 5 | 1 | 1,14 | 1,40 | 2,04 | 2,26 |  |
| Mm_miR-185 | 8 | 1 | 1,82 | 1,62 | 2,10 | 2,23 | 3,15 |
|  | 5 | 1 | 1,63 | 1,79 | 2,42 | 3,41 |  |
| Mm_miR-187 | 8 | 1 | 1,90 | 2,07 | 2,45 | 2,06 | 3,25 |
|  | 5 | 1 | 1,33 | 1,65 | 2,13 | 2,59 |  |
| Mm_miR-193 | 8 | 1 | 1,29 | 1,27 | 1,70 | 1,89 | 3,00 |
|  | 5 | 1 | 1,58 | 1,80 | 2,08 | 2,75 |  |
| Mm_miR-193b | 8 | 1 | 1,46 | 1,63 | 2,15 | 2,57 | 3,75 |
|  | 5 | 1 | 1,64 | 1,49 | 1,85 | 2,60 |  |
| Mm_miR-194 | 8 | 1 | 1,77 | 1,93 | 1,98 | 2,13 | 3,21 |
|  | 5 | 1 | 1,57 | 2,11 | 1,79 | 3,12 |  |
| Mm_miR-199a-3p | 8 | 1 | 2,21 | 2,83 | 3,71 | 3,37 | 4,67 |
|  | 5 | 1 | 2,13 | 2,45 | 3,03 | 4,73 |  |
| Mm_miR-199a | 8 | 1 | 2,08 | 2,61 | 3,15 | 3,04 | 4,71 |
|  | 5 | 1 | 2,27 | 3,01 | 4,10 | 6,04 |  |
| Mm_miR-199b* | 8 | 1 | 2,42 | 2,51 | 3,49 | 3,10 | 5,47 |
|  | 5 | 1 | 1,90 | 2,77 | 4,55 | 4,90 |  |
| Mm_miR-214 | 8 | 1 | 1,49 | 1,95 | 2,02 | 1,92 | 2,39 |
|  | 5 | 1 | 1,73 | 2,39 | 2,40 | 3,38 |  |
| Mm_miR-214* | 8 | 1 | 1,74 | 1,11 | 1,74 | 1,46 | 2,36 |
|  | 5 | 1 | 1,40 | 1,12 | 1,70 | 2,00 |  |
| Mm_miR-218 | 8 | 1 | 2,82 | 3,08 | 3,75 | 2,43 | 4,53 |
|  | 5 | 1 | 1,55 | 1,99 | 2,17 | 2,76 |  |
| Mm_miR-299 | 8 | 1 | 1,48 | 1,55 | 2,22 | 1,91 | 1,91 |
|  | 5 | 1 | 1,20 | 1,39 | 2,71 | 2,72 |  |
| Mm_miR-320 | 8 | 1 | 1,47 | 2,30 | 2,24 | 2,58 | 3,10 |
|  | 5 | 1 | 1,38 | 1,88 | 2,53 | 3,08 |  |
| Mm_miR-339 | 8 | 1 | 1,57 | 2,28 | 2,50 | 2,31 | 3,32 |
|  | 5 | 1 | 1,34 | 1,55 | 2,03 | 2,61 |  |
| Mm_miR-379 | 8 | 1 | 1,34 | 1,42 | 2,47 | 1,52 | 2,16 |
|  | 5 | 1 | 1,98 | 1,19 | 2,17 | 2,90 |  |
| Mm_miR-411 | 8 | 1 | 1,55 | 1,49 | 2,55 | 2,12 | 4,08 |
|  | 5 | 1 | 1,47 | 1,17 | 3,62 | 3,27 |  |
| Mm_miR-421 | 8 | -1 | -1,50 | -2,50 | -2,07 | -2,63 | -1,82 |
|  | 5 | -1 | -1,22 | -2,80 | -2,07 | -2,70 |  |
| Mm_miR-422b | 8 | 1 | 4,49 | 1,72 | 1,95 | 2,43 | 2,14 |
|  | 5 | 1 | 2,19 | -1,02 | 1,26 | 1,66 |  |
| Mm_miR-455 | 8 | 1 | 1,33 | 2,87 | 3,19 | 3,68 | 4,46 |
